# Shaping and coordinating the implementation science agenda for injectable cabotegravir for PrEP: the role of the Biomedical Prevention Implementation Collaborative (BioPIC)

**DOI:** 10.1002/jia2.26094

**Published:** 2023-07-13

**Authors:** Mitchell Warren, Wawira Nyagah, Catherine Verde Hashim, Michelle Rodolph, Robin Schaefer, Heather‐Marie A Schmidt, Rachel Baggaley

**Affiliations:** ^1^ AVAC New York USA; ^2^ AVAC Nairobi Kenya; ^3^ AVAC London UK; ^4^ Global HIV, Hepatitis, and STI Programmes World Health Organization Geneva Switzerland; ^5^ UNAIDS Regional Office for Asia and the Pacific Bangkok Thailand

**Keywords:** cabotegravir, HIV, implementation science, PrEP, prevention, product introduction

## Abstract

**Introduction:**

Data from two randomized controlled trials (RCTs) showed that injectable cabotegravir (CAB) for pre‐exposure prophylaxis (PrEP) was efficacious in reducing HIV acquisition. The US Food and Drug Administration approved CAB for PrEP in December 2021; Australia in August 2022; Zimbabwe in October 2022; South Africa in November 2022; Malawi in March 2023; and regulatory approvals are being sought in additional countries. The World Health Organization (WHO) recommended CAB be offered to people at substantial risk of HIV in July 2022. However, implementation experience beyond RCTs is limited. As countries consider CAB implementation, questions remain regarding delivery and involvement of populations excluded from the trials. A coordinated approach is needed to ensure these are addressed and CAB can be introduced in low‐ and middle‐income countries in timely, acceptable and effective ways.

**Discussion:**

Beginning in 2018, the Biomedical Prevention Implementation Collaborative (BioPIC) convened over 100 global health experts to develop a comprehensive introduction strategy for CAB. Using this roadmap, country landscaping for CAB introduction and lessons from oral PrEP implementation, AVAC and WHO co‐convened 50 researchers, donors, implementers and civil society in September 2021 to: (1) identify questions and evidence gaps related to CAB across contexts and partners; (2) define the implementation science agenda; and (3) agree on mechanism(s) for future coordination. As a result, CAB‐related questions were identified, including: defining optimal and feasible HIV testing strategies that expand access; delivery models; integration with a range of services, including family planning and antenatal care; and embedding CAB in demand generation for HIV prevention choices. Through convenings and mapping of implementation research, BioPIC identified gaps in populations, geographies and delivery approaches.

**Conclusions:**

The introduction strategy refined by BioPIC lays the groundwork for future HIV prevention products. Ongoing policy and implementation dialogue is critical to accelerate the design of CAB implementation studies that adequately address priority knowledge gaps. Additional long‐acting HIV prevention products may be available over the next 5 years, increasing choice, but potentially making delivery and stakeholder engagement more complex. Ongoing coordination with WHO will accelerate the adoption of evidence‐based policies and wide‐scale implementation, and lessons from BioPIC can inform introduction processes for long‐acting HIV prevention products.

## 1 INTRODUCTION

Injectable cabotegravir (CAB) is an integrase strand‐transfer inhibitor (INSTI) class antiretroviral. For use as HIV pre‐exposure prophylaxis (PrEP), it is administered to people who do not have HIV at a dose of 600 mg, intramuscularly, 4 weeks apart for the first two injections and every 8 weeks thereafter.

Data from the HIV Prevention Trials Network (HPTN) 083 and 084 randomized controlled trials (RCTs) in 2020 indicated that CAB for PrEP is safe and efficacious in reducing the risk of HIV acquisition in multiple populations. HPTN 083 results suggest an estimated 66% relative reduction in HIV acquisition risk among cisgender men and transgender women who have sex with men [1] and HPTN 084 results suggest an 88% reduction among cisgender women compared with oral PrEP [2]. These trials have now transitioned from blinded to open‐label studies in which all trial participants are offered the choice between injectable and oral PrEP.

US regulatory approval in December 2021 paves the way for introduction in the United States. Australia approved the product in August 2022, the Zimbabwean regulatory agency approved it in October 2022, while South Africa approved it in November 2022 and Malawi in March 2023. Additional regulatory approvals are being sought in a range of countries, including several low‐ and middle‐income countries (LMICs) [3]. In July 2022, the World Health Organization (WHO) recommended that injectable CAB may be offered as an additional HIV prevention option to people at substantial HIV risk [4].

However, implementation experience beyond the RCTs is limited. The approvals and guidelines have brought increasing momentum, discussion and concern around what is happening to ensure the introduction of CAB for PrEP maximizes its prevention potential, including whether the initial implementation science projects will answer critical remaining questions. The experiences of the first decade of oral PrEP implementation show the impact of delays in delivery and inequity in access [5]. As countries consider CAB introduction, there remain important implementation science questions regarding the delivery of CAB—notably regarding HIV testing and HIV drug resistance—and the involvement of populations not included in the trials [6, 7] that need to be answered in order to translate the strong efficacy seen in the clinical trials into public health impact. A coordinated implementation science approach is needed to ensure these are addressed and that CAB can be introduced at scale and with equity, especially in LMICs, in timely, acceptable and effective ways.

With an accelerated timeline for planning and preparing to introduce CAB prompted by the early, positive results of the two pivotal RCTs, and the progress of other long‐acting HIV prevention products through the pipeline, AVAC and WHO, through the Biomedical Prevention Implementation Collaborative (BioPIC), jointly convened key stakeholders to strategize about implementation projects and other planning for product introduction.

Established in 2018 by AVAC and the Clinton Health Access Initiative (CHAI) with funding from the Bill and Melinda Gates Foundation, the BioPIC takes an innovative, collaborative approach to support rapid, successful introduction of HIV prevention products to ensure activities are well‐designed, well‐timed and well‐funded to meet the needs of global and country decision‐makers. It is made up of over 100 HIV prevention experts, including civil society representatives, donors, researchers, policymakers, normative agencies and implementers from over 20 countries, and is now serving as the secretariat of the Coalition to Accelerate Access to Long‐Acting PrEP.

## 2 DISCUSSION

Identifying feasible, affordable and acceptable strategies for delivering injectable CAB for PrEP is critical to ensuring that it—and other emerging HIV prevention tools—offer greater options to individuals who may benefit from using antiretroviral drugs for prevention and can have an impact on the epidemic.

Beginning in 2018, BioPIC convened over 100 global health experts to develop a comprehensive introduction strategy for CAB, including an adaptable framework for HIV prevention product introduction [8]. Using this framework alongside country‐specific landscape analyses of demand and supply issues for CAB introduction and lessons from oral PrEP implementation [5], AVAC and WHO co‐convened researchers, donors, implementers and civil society through a series of virtual thinks tanks to: (1) identify common questions and evidence gaps related to CAB across contexts and partners; (2) define the implementation science agenda for CAB that can help inform and, hopefully, accelerate programme scale; and (3) agree on mechanism(s) for future coordination. Think Tank topics have included: Product Introduction/Implementation Project Planning for Next‐Generation PrEP (22–23 September 2021); HIV Testing and Injectable CAB for PrEP Introduction: What are the implications for HIV prevention scale‐up and the HIV response? (3 December 2021); Modelling the Impact of Injectable CAB for PrEP on Drug Resistance (7 March 2022); Coordinating Implementation Science for CAB for PrEP (21 April 2022); Generating Demand for HIV Biomedical Prevention in the Era of Choice (7–8 June 2022); Coordinating Implementation Science for CAB for PrEP: Focus on Delivery Models (23 June 2022); Coordinating Implementation Science for CAB for PrEP: Focus on Research and Surveillance during Pregnancy and Breastfeeding (2 November 2022); Shaping and Strengthening Markets for a New Era of Choice‐based HIV/SRH Programming: Lessons from HIV & FP Product Introduction (1 March 2023); and Bridging from the HPTN 083 and 084 Open Label Extensions to Implementation (14 March 2023) [9].

As a result, CAB‐related research and implementation questions were identified, which fell into four primary categories: populations, demand creation, delivery and data. Specifically, identified research questions included: defining optimal and feasible HIV testing strategies that balance the need for affordable and feasible testing with the need to detect infections early to prevent potential INSTI resistance; delivery models; integration with a range of services, including for family planning and antenatal care; and how to embed CAB in overall demand generation for HIV prevention choices.

Through convenings and mapping of proposed implementation science projects, BioPIC identified and encouraged researchers to fill gaps in these issues. This process began by identifying priority implementation science planning issues to ensure robust, strategic and informative demonstration and introduction projects of injectable CAB for PrEP.

### 2.1 Populations

Future studies need to generate information for implementation among diverse populations and geographies, looking especially beyond adolescent girls and young women in sub‐Saharan Africa, the focus of many PrEP programmes to date and need to explore concerns, questions, service preferences and other issues through implementation projects when feasible or other research and community‐based approaches. There is a need to ensure that all regions and “missing populations” from the RCTs, such as trans and gender‐diverse people, people who inject drugs, sex workers and adolescents are included in some aspects of this implementation research. There are also remaining questions regarding the safety of CAB during pregnancy and breastfeeding as well as potential interactions between CAB and gender‐affirming hormones (although recent data suggest that there is limited impact of oestradiol‐based gender‐affirming hormones on CAB concentrations) [10]. These projects will need to balance messaging and services tailored to specific users and user groups with normalizing use and avoiding stigma.

### 2.2 Demand creation—for CAB, for PrEP and for HIV prevention generally

As projects are designed, potential users and user groups should be defined by behaviours rather than risk, reframing injectable CAB—and other PrEP products—affirmatively by stressing benefits, such as the potential for reduced anxiety, increased connection with partners and enjoyment rather than “risk reduction.” Implementation projects should invest in robust partnerships with community and potential users early and continuously, using multiple approaches—advisory groups, paid support to community‐based organizations, media engagement, creation and dissemination of resource and marketing materials—to involve civil society from the beginning as experts in designing services, communications strategies and materials, and outreach. While injectable CAB is the newest option, it is not the only one, and projects should ensure that messaging emphasizes HIV prevention choices (including also the dapivirine vaginal ring) and does not play one PrEP product against another.

### 2.3 Delivery

Implementation projects should be designed to explore and anticipate a range of models and approaches, such as the role of HIV testing at initiation and on an ongoing basis, integration with other services, community delivery (including through pharmacies), task sharing and involvement of peer and lay providers in delivery. These projects can be located within existing services for HIV prevention, family planning, primary healthcare and others to explore how people choose and potentially switch among different options like oral PrEP, CAB, the dapivirine vaginal ring and condoms, what typical patterns of use look like and how to best support effective adherence. There are important lessons and examples from a range of health services and systems beyond PrEP. For example, contraceptive programmes have long experience with offering clients a choice of methods and in managing switching and “discontinuation,” and injectable contraception has clear parallels with CAB. Now that multiple antiretroviral (ARV)‐based prevention options may be available, projects need to monitor, understand and support use across the product spectrum, including initial product uptake, continuation and possible switching between methods for the first time. Also, these projects should be designed from the outset to be scalable, pragmatic and sustainable.

### 2.4 Data

Upcoming projects should include specific outcome measures that explore how people choose among different prevention options, as well as patterns of starting/stopping and switching, and their reasons for doing so. This will require agile monitoring and evaluation systems and indicators that can accurately monitor use patterns in programmes, differences across service delivery and demand creation models, and costs. Given concerns for potential HIV drug resistance, it is essential to establish robust, feasible monitoring systems for resistance in PrEP programmes. Effective surveillance systems for pregnancy outcomes will also be critical to generate evidence on the safety of CAB for PrEP during pregnancy and breastfeeding. Coordinating and sharing information among projects will be needed to develop an evidence base and clear strategy to actively cultivate interest and address questions from governments that may have limited capacity for and/or interest in investing in new products, including cost and cost‐effectiveness.

Research approaches and outcomes will likely vary by context, population and other factors, so it will be important that implementation science is seamlessly part of scale‐up rather than a separate step. In the context of oral PrEP programmes, there was a reluctance to initiate large‐scale programmes before the implementation science projects were completed. Given the epidemiologic need and the potential of this new prevention option, it is possible to use real‐time data to inform and drive accelerated scale‐up with efforts to identify effective strategies for specific populations and geographies, planning in parallel to answer questions and build programmes, rather than seeing implementation science and scaled programmes as sequential. Implementation research will need to be adapted to various contexts, but for the most part drive towards similar outcomes (Table 1). These outcomes would evaluate the overall effectiveness and impact of both individual and cumulative HIV prevention products on the epidemic, document actual acceptability, use patterns and challenges in “real world” conditions and compare the effectiveness of different delivery, demand creation and community engagement models. Standardizing outcome measures across projects and partners can help to produce more comparative and generalizable data to drive decision‐making and identify what strategies can and should be taken to scale.

**Table 1:** Potential outcome measures for implementation science research on injectable cabotegravir (CAB) for pre‐exposure prophylaxis (PrEP)

Category	Outcomes
Uptake, use and impact	Where feasible, disaggregate outcomes by population, age and gender: Proportion of eligible individuals initiating CAB Proportion of new PrEP (PrEP naïve) CAB enrolments versus switches from other PrEP products to CAB Proportion of individuals who seroconvert while using CAB Proportion of individuals who acquire drug‐resistant HIV infections due to PrEP exposure versus those who acquire transmitted drug resistance Proportion of individuals who are initiated on CAB after acquiring HIV Proportion of individuals who stop using CAB and switch to another prevention method, who stop and use another prevention method to cover the tail, who stop without using another HIV prevention method; reasons for doing so Reduction in HIV incidence among CAB users; number of HIV infections averted
Cost‐effectiveness	Delivery and commodity costs of CAB Cost of PrEP delivery by model typology Feasibility and health facility readiness for CAB implementation Cost‐effectiveness and cost‐utility of CAB in different implementation scenarios, including when CAB is prioritized to “high‐risk” individuals or all individuals who could benefit from PrEP and comparisons to other PrEP options. Cost‐effectiveness of different HIV testing approaches Cost of preparing health facilities for CAB implementation
Collateral impacts	Impact of delivering CAB within family planning clinics on contraceptive uptake and adherence Impact of use of CAB on sexual behaviour and sexually transmitted infections (STI) incidence Impact of CAB availability and use on overall HIV prevention coverage Impact of delivery of CAB on uptake of a range of primary healthcare services, including ante‐natal and postnatal care attendance and uptake of sexual and reproductive health services Changes in reported quality of life, depression or anxiety and self‐efficacy/empowerment
Community and end‐user engagement	Increased community awareness and education of CAB as a prevention option Increased end‐user and community participation in CAB implementation research, delivery, monitoring and demand generation

Through this process, a CAB for PrEP Implementation Study Tracker [11] was established to track all currently known partner activities relating to landscaping, product introduction, introduction studies and implementation research. These studies are mapped against the Implementation Science Questions for CAB for PrEP framework in Table 1 [12] to monitor for any gaps, overlaps and/or opportunities for collaboration, and BioPIC and WHO continue to convene researchers to facilitate sharing implementation plans and information, to drive innovation and collaboration, and to coordinate among projects for different geographies, populations and donors.

## 3 CONCLUSIONS

With over 25 implementation research studies for injectable CAB for PrEP already being at different stages of planning and preparation [8] and as programmes shift towards choice‐based models for HIV prevention, this is a critical time to correct for missed opportunities with oral PrEP rollout, and ensure more effective coordination across geographies, populations and projects. This work needs to run in parallel with ongoing advocacy related to accelerate access to the product and reduce the cost of the product, both significant barriers to scale and impact.

Oral PrEP was first shown to be safe and effective in 2010 and first approved for use in the United States in 2012, but the field moved slowly—and now 10 years later, only approximately 3 million people have initiated the use of this option and approximately 1.6 million current users by the end of 2021, far slower than the 2020 target of 3 million, far short of the new 2025 target of 10 million PrEP users and a tiny fraction of the estimated number of people who need it and could benefit from it. There are significant questions about how to deliver injectable CAB for PrEP, therefore, coordinated partnerships and bold action are needed urgently.

The implementation science agenda refined by BioPIC lays the groundwork for future HIV prevention products. Ongoing policy and implementation dialogue, including with civil society, is critical to accelerate the design of CAB implementation studies that adequately address priority knowledge gaps. Additional long‐acting products may be available over the next 5 years, increasing choice, but potentially making delivery and stakeholder engagement more complex. Ongoing coordination with WHO will also accelerate the adoption of evidence‐based policies and wide‐scale implementation. While any one project may or may not be a success on its own, systematically monitoring and analysing how the introduction and delivery of CAB for PrEP across the evolving ecosystem affects key individual‐level and public health‐level questions identified in this process can contribute to building a sustainable platform for HIV prevention. The need for enhanced coordination has never been greater and lessons from BioPIC can inform introduction processes not only for CAB but also for other products.

## COMPETING INTERESTS

The authors declare that they have no competing interests.

## AUTHORS’ CONTRIBUTIONS

MW drafted the initial version of the manuscript. All authors reviewed and revised the manuscript. All authors approved the final version of the manuscript.

## FUNDING

MW, WN and CVH were supported by a grant from the Bill & Melinda Gates Foundation, and MW was also supported by the Coalition to Accelerate and Support Prevention Research (CASPR), made possible by the generous support of the American people through the US President's Emergency Plan for AIDS Relief (PEPFAR) and the US Agency for International Development (USAID). MR, RS, HS and RB all work for the World Health Organization which receives grants from the Bill & Melinda Gates Foundation, USAID and Unitaid for their work on PrEP.

## DISCLAIMER

The authors alone are responsible for the views expressed in this article and they do not necessarily represent the views, decisions or policies of the institutions with which they are affiliated.

## Data Availability

Data sharing is not applicable to this article as no datasets were generated or analysed during the current study.
